# Fake news reminders and veracity labels differentially benefit memory and belief accuracy for news headlines

**DOI:** 10.1038/s41598-022-25649-6

**Published:** 2022-12-17

**Authors:** Paige L. Kemp, Vanessa M. Loaiza, Christopher N. Wahlheim

**Affiliations:** 1 Department of Psychology, University of North Carolina at Greensboro, 296 Eberhart Building, P. O. Box 26170, Greensboro, NC 27402-6170 USA; 2 Department of Psychology, University of Essex, Colchester, UK

**Keywords:** Psychology, Human behaviour

## Abstract

Fake news exposure can negatively affect memory and beliefs, thus sparking debate about whether to repeat misinformation during corrections. The once-prevailing view was that repeating misinformation increases its believability and should thus be avoided. However, misinformation reminders have more recently been shown to enhance memory and belief accuracy. We replicated such reminder benefits in two experiments using news headlines and compared those benefits against the effects of veracity labeling. Specifically, we examined the effects of labeling real news corrections to enhance conflict salience (Experiment 1) and labeling fake news on its debut to encourage intentional forgetting (Experiment 2). Participants first viewed real and fake news headlines with some fake news labeled as false. Participants then saw labeled and unlabeled real news corrections; labeled corrections appeared alone or after fake news reminders. Reminders promoted the best memory and belief accuracy, whereas veracity labels had selective effects. Correction labels led to intermediate memory and belief accuracy, whereas fake news labels improved accuracy for beliefs more than memory. The extent that real and fake news details were recalled together correlated with overall memory and belief differences across conditions, implicating a critical role for integrative encoding that was promoted most by fake news reminders.

Fake news refers to stories including verifiably false information presented as true. Although fake news has been around for centuries, it recently gained widespread attention when misinformation about the 2016 and 2020 US Presidential elections, the UK Brexit Referendum, and the coronavirus disease 2019 (COVID-19) spread across social media platforms^1^. Fake news exposure can have negative consequences for people and societies, such as when COVID-19 misinformation diminished the willingness to vaccinate and recommend vaccination^2^. These and other threats to public health and democracy emphasize the importance of identifying effective correction methods. Reminding people of real-world fake news before correcting it can substantially enhance memory and belief accuracy^3^. Additionally, veracity labels about the ground truth of news headlines may reduce false beliefs and sharing behaviors^4^. However, we know virtually nothing about how updating memory and beliefs for factual information compares for correction methods using fake news reminders and veracity labels. The present study addressed this issue by comparing memory and belief accuracy for real news headline details when corrections included fake news reminders, only veracity labels for corrections, or only veracity labels for fake news.

Predictions about memory and belief accuracy under these correction methods can be derived from perspectives on misinformation corrections proposing key roles for familiarity and integration mechanisms. A robust finding that has inspired these existing perspectives originates from studies of the *continued influence effect*. This effect occurs when retracting misinformation does not completely eliminate its influence on event comprehension and reasoning^5,6^. This effect may persist when retractions include misinformation, thus increasing misattributions of its familiarity when contextual details are not recollected^7,8^. This *familiarity-backfire* view was originally proposed to account for the finding that retractions repeating misinformation increased misinformation-based behavioral intentions after a delay^9^. According to this view, memory and belief accuracy for real news headlines that correct fake news should be better when only veracity labels are provided than when fake news reminders appear before real news corrections because reminders would promote fake news familiarity that could backfire.

Although the backfire view has enjoyed popularity^10,11^, many studies have failed to find this effect^12,13^. For example, in a study of knowledge revision, beliefs in retracted myths were less sustained relative to affirmed facts after a 3-week delay, but a true backfire effect was not observed because post-retraction beliefs did not regress beyond baseline beliefs^14^. Additionally, retractions featuring an explicit misinformation reminder reduced the continued influence effect more than retractions without a reminder^15^. According to *conflict salience* accounts of mental-model updating, the misinformation repetition fostered co-activation of the erroneous and correct information, enabling conflict detection and updating of event models and beliefs^16,17^. This view is compatible with the assertion that detecting conflict between events is necessary to facilitate memory and belief updating^18–20^. Moreover, these findings show how repetition-induced familiarity does not always backfire, thus undermining the prior recommendation to avoid reminders of misinformation^8^.

In contrast with predictions from the familiarity backfire view, a recent study showed clear evidence that reminders of fake news can enhance the accuracy of memory for and beliefs in real news corrections^3^. Participants first read news headlines of unclear veracity then read headlines that affirmed real news and corrected fake news. Some of the corrections were preceded by a fake news reminder, while others were not. Similar to earlier findings^15^, reminders improved memory and belief accuracy for real news headlines. These benefits were associated with real news details being recalled more often when fake news details were also recalled. According to the *integrative encoding* view, reminders led both fake and real news detail to be co-activated in working memory. This provided the opportunity for those details to be encoded together into an integrated representation that included information about their veracity and relationship to one another^16,17,21^. However, a key limitation was that veracity labels appeared with real news corrections that followed fake news reminders, but there was no contrast condition with only veracity-labeled real news corrections. Thus, the contributions of conflict salience and integrative encoding to reminder-induced benefits could not be separated. If integrative encoding contributes beyond the salience from veracity labels, then memory and belief accuracy should be higher when comparing a fake news reminder condition with a condition including only veracity-labeled corrections without reminders.

An additional objective of the current study was to compare the efficacy of fake news reminders to another veracity-labeling method that has yet to be explored. Studies have explored how correction formats influence memory, showing that ordering of myths and facts has no effect^22^, but labels refuting fake news are more effective when they appear after instead of before or during fake news exposure^23^. Related to these findings, memory and belief updating may depend on the extent to which people can disregard veracity-labeled fake news immediately after it appears. This idea is supported by work on directed forgetting showing that under specific circumstances, memory for recently learned information is better when participants are instructed to forget earlier-learned information that can serve as a source of proactive interference^24,25^.We addressed this issue here by comparing memory and belief accuracy when fake news is labeled on its debut compared to when it is only labeled when appearing as a reminder. The integrative encoding account predicts that fake news reminders will lead to better memory and belief updating by promoting co-activation, whereas a differentiation view from the context-dependent memory literature^26^ predicts that real news details should suffer less proactive interference when co-activation is prevented. However, labeling fake news on its debut could make it more distinctive and available for integrative encoding.

The benefits of fake news reminders attributed to integrative encoding have been accounted for by a verbal theory proposing that integration enhances recollection-based retrieval of competing details and their relationship^21^. We evaluated this claim here using a hierarchical Bayesian Multinomial Processing Tree (MPT) approach. MPT modeling can describe the cognitive processes underlying cued recall responses^27^. We used this approach to estimate the contributions of recollection of headlines’ veracity and acontextual familiarity of headline topics to final real news recall. Based on dual-process models of memory^28,29^ and reasoning^30–32^, we assumed that recalling corrections of fake news required recollection to override the familiarity of fake news.

## The present study

We conducted two experiments to examine whether the benefits of presenting reminders of fake news immediately before veracity-labeled real news corrections would extend to naturalistic news headline stimuli including both images and text. We also compared the efficacy of reminder-based corrections against veracity-labeled real news corrections without reminders (Experiment 1) and veracity-labeled fake news on its debut (Experiment 2). These comparisons were intended to illuminate the mechanisms underlying fake news reminder effects. Labeling only real news should increase its saliency and signal participants to prioritize remembering it, whereas labeling only fake news could encourage participants to disregard it or make it more distinctive. Regardless of the precise effects of veracity labeling, fake news reminders should better promote integrative encoding by increasing opportunities for co-activation more than veracity labels alone.

**Figure 1:**
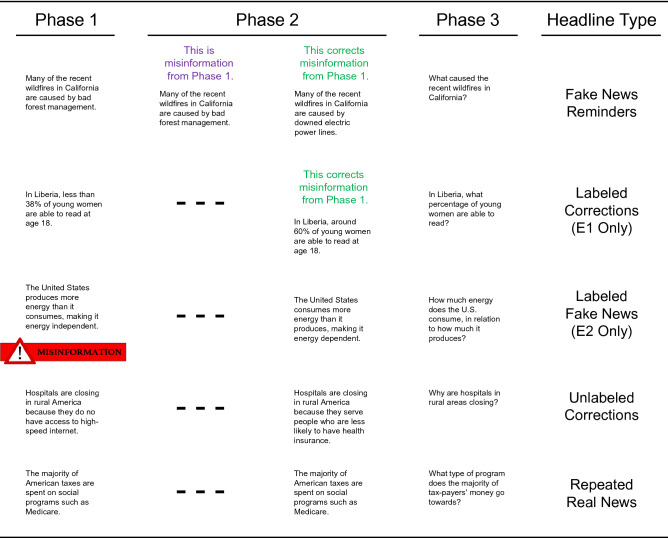
Illustration of the experimental designs. Phase 1 included real and fake news headlines, most of unclear veracity. Phase 2 included corrections of fake news and exact repetitions of real news headlines with the same picture and wording as in Phase 1. The labeled corrections (second row) only appeared in Experiment 1 (E1), and the labeled fake news (third row) only appeared in Experiment 2 (E2). Phase 3 included images that appeared with the headlines from the prior phases and questions about key details that were corrected when headlines appeared as fake news in Phase 1 and corrections in Phase 2. Images that appeared in the experiments are not displayed here due to copyright issues.

We tested this hypothesis using a procedure in which participants first read real and fake news headlines from the internet and indicated their familiarity with and belief in each headline (Phase 1). Participants then read real news headlines that verified real news and corrected fake news from Phase 1 (Phase 2). Finally, participants were given a cued recall test including images from the original headlines. Below the headlines were questions about details that were either repeated across phases or were corrected in the second phase. Participants attempted to recall both real and fake news details (when applicable) and indicated their belief in what they recalled as real news (Phase 3). Fake news reminders appeared before some real news headlines labeled as corrections in Phase 2. For other headlines, real news headlines were labeled as corrections in Phase 2 (Experiment 1) and fake news headlines were labeled as misinformation in Phase 1 (Experiment 2). Real news headlines also appeared in Phase 2 as unlabeled corrections of fake news and repetitions of real news from Phase 1. Figure 1 illustrates how headlines appeared in each phase across these within-subjects conditions.

Based on prior findings showing that labels alone can improve memory and belief accuracy^23,33^, we expected that labeling only real news corrections or only fake news would improve memory and belief accuracy by providing details that can be recollected to accept (for real news) and reject (for fake news) headlines. However, presenting reminders before corrections can enhance memory and reasoning beyond labels alone^15^. We therefore expected that including fake news reminders before real news corrections would lead to the most accurate memory and beliefs by promoting integrative encoding of representations that best support recollection^3,15,16,34,35^. To the extent that memory and belief accuracy differ across correction methods, we expected process estimates from the MPT models to show corresponding differences in the contributions of recollection. It was unclear whether familiarity would contribute differently across conditions as it could promote correct recall or misattributions of fluently recalled fake news^36^.

## Results and discussion

We performed hypothesis tests using mixed effects models including by-participant and by-item random intercept effects to account for those sources of variability. We describe the statistical methods for all measures in the Supplementary Information (henceforth SI) “Introduction” (i.e., SI1). We also describe additional exploratory analyses that were not central to the goals of the present study in SI7. In Phase 1, the baseline measures of familiarity and beliefs indicated that participants perceived real news headlines as more familiar (SI2.1) and believable (SI2.2) than fake news headlines. In Experiment 2, participants believed veracity-labeled fake news headlines far less than all the other unlabeled headlines.

### Fake news reminders enhanced real news recall more than labeling corrections

**Figure 2:**
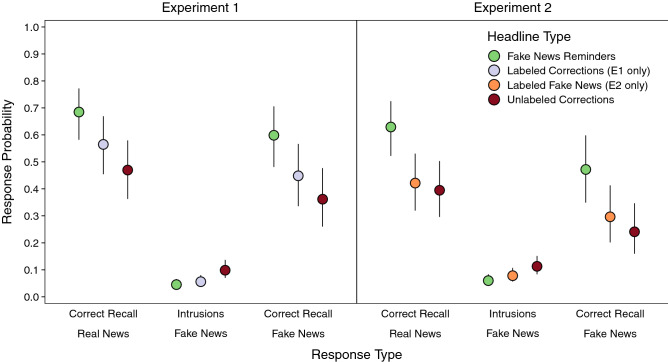
Probabilities of real news recall, intrusions of fake news, and fake news recall in Phase 3 for each correction headline type condition. Points are probabilities estimated from mixed effects models; error bars are 95% confidence intervals.

Table 1 displays the complete model results for all analyses of cued recall in Phase 3. Participants recalled real news corrections of fake news in Phase 3 most accurately when fake news reminders had appeared in Phase 2 (Fig. 2). Experiment 1 showed significantly higher real news recall when fake news reminders preceded corrections regardless of whether corrections alone were labeled or unlabeled, smallest *z* ratio = 5.32, *p* < 0.001. Additionally, real news recall was significantly higher for labeled than unlabeled corrections, *z* ratio = 4.06, *p* < 0.001. Experiment 2 showed significantly higher real news recall when fake news reminders immediately preceded corrections than in the other correction conditions, smallest *z* ratio = 8.91, *p* < 0.001. Real news recall for unlabeled corrections did not differ based on whether veracity labels accompanied fake news in Phase 1, *z* ratio = 1.19, *p* = 0.63. Finally, correct recall for real news that repeated from Phase 1 to Phase 2 (Experiment 1: 0.76 [95% CI 0.67, 0.83]; Experiment 2: 0.70 [95% CI 0.60, 0.78]; not pictured) was significantly higher than for all correction conditions, smallest *z* ratio = 3.28, *p* < 0.01. Collectively, these results suggest that using fake news reminders to encourage the integration of real and fake news promoted real news recall more than increasing conflict saliency for corrections or encouraging participants to disregard fake news with veracity labels.

**Table 1:** Model results for real news recall, intrusions of fake news, and fake news recall in Phase 3.

Analysis	Effect	Experiment 1			Experiment 2		
		χ^2^	*df*	*p*	χ^2^	*df*	*p*
Overall real news recall	Headline type	186.74	3	< 0.001	245.57	3	< 0.001
Overall intrusions of fake news	Headline type	48.68	2	< 0.001	34.60	2	< 0.001
Overall fake news recall	Headline type	97.04	2	< 0.001	113.31	2	< 0.001
Conditional real news recall	Headline type	13.40	2	< 0.01	34.15	2	< 0.001
	Classification	622.38	2	< 0.001	635.69	2	< 0.001
	Headline type × classification	3.14	4	= 0.54	4.06	4	= 0.41
Conditional intrusions of fake news	Headline type	7.17	2	= 0.03	4.92	2	= 0.09
	Classification	84.77	1	< 0.001	48.66	1	< 0.001
	Headline type × classification	6.64	2	= 0.04	6.44	2	= 0.04

**Figure 3:**
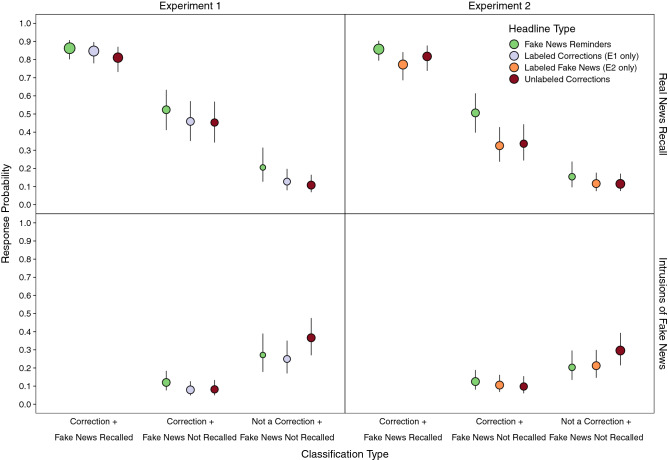
Probabilities of real news recall and intrusions of fake news conditioned on correction classifications for each correction headline type condition. Points are probabilities estimated from mixed effects models; error bars are 95% confidence intervals. Point sizes indicate for each cell the relative proportion of observations, which are also displayed in Supplementary Table S4. Values are not displayed for intrusions for classified corrections when fake news was recalled due to sparse observations.

The results above correspond to the data visualized in Fig. 2 (for overall recall) and Fig. 3 (for conditional recall).

### Veracity labels reduced intrusions of fake news

More information about differences in memory accuracy across correction methods can be gleaned from examining intrusions of fake news from Phase 1 during recall of real news from Phase 2 (Fig. 2). Memory accuracy on this measure is higher when intrusion rates are lower, indicating fewer memory misattributions. Both experiments showed that labeling corrections of fake news, regardless of whether fake news reminders appeared in Phase 2, led to lower intrusion rates than presenting headlines without labels. Intrusions were significantly higher for unlabeled corrections than for all other corrections, smallest *z* ratio = 3.55, *p* < 0.01, and were not significantly different among the other corrections, *z* ratio = 2.29, *p* = 0.06. Thus, veracity labels uniformly reduced memory misattributions.

### Reminders enhanced fake news recall more than veracity labels

Real news may be better remembered when the details become integrated with the fake news they corrected. We first examined potential associations between fake and real news recall by characterizing the accessibility of fake news across correction conditions (Fig. 2). Both experiments showed that providing fake new reminders before labeled corrections led to significantly higher fake news recall than all other corrections, smallest *z* ratio = 6.24, *p* < 0.001. Additionally, only labeling corrections (Experiment 1) or fake news (Experiment 2) led to significantly higher fake news recall than presenting corrections without labels, smallest *z* ratio = 2.88, *p* = 0.01. These results suggest that repeating fake news as reminders made those headlines most memorable, labeling corrections promoted retrieval practice of fake news when participants thought about what was corrected, and labeling fake news made it more distinctive, despite participants being told to disregard those headlines.

### Reminders promoted integrative encoding over veracity labels alone

We further examined the role of fake news retrieval and integrative encoding during encoding of corrections in memory accuracy for the three correction types in each experiment by computing real news recall conditioned on fake news recall and correction classifications. We created three categories based on combinations of correction classifications and fake news recall (Fig. 3). The first two categories included accurately classified corrections that varied based on whether fake news was subsequently recalled. *Correction* + *Fake News Recalled* refers to headline topics for which participants remembered there was a correction and could recall the fake news detail. *Correction* + *Fake News Not Recalled* refers to headline topics for which participants remembered there was a correction and could not recall the fake news detail. *Not a Correction* + *Fake News Not Recalled* refers to headline topics for which participants did not remember there was a correction and thus did not recall the fake news detail. Trial proportions corresponding to point sizes in Fig. 3 are shown in Supplementary Table S4.

Based on our prior findings^3,34,37^, we reasoned that integration differences across correction types could be inferred from differences in real news recall probabilities conditioned on fake news also being recalled. In both experiments, real news recall (Fig. 3, top panels) was significantly higher for accurately classified corrections accompanied by fake news recall than for other classification types, smallest *z* ratio = 15.01, *p* < 0.001, and for accurately classified corrections when fake news was not recalled than corrections that were inaccurately classified, *z* ratio = 11.21, *p* < 0.001. Taken with the differences in fake news recall across headline types described above, these findings suggest that real news recall was facilitated to the extent that corrections promoted the co-activation of fake and real news, thus supporting subsequent recollection.

### Fake news intruded more for inaccurately classified corrections

We also examined the extent to which remembering corrections was associated with intrusion reduction, as shown before^3,34^. Note that we did not include classifications for which fake news was recalled because intrusions of fake news were redundant responses that seldom occurred. Both experiments showed that intrusions of fake news (Fig. 3, bottom panels) were significantly lower for accurately than inaccurately classified corrections. Significant interactions showed that when corrections were inaccurately classified, there were significantly more intrusions for unlabeled than labeled corrections in Experiment 1, *z* ratio = 2.88, *p* = 0.01, and unlabeled than both other corrections in Experiment 2, smallest *z* ratio = 2.58, *p* = 0.03. These results suggest that remembering that a topic was corrected counteracted familiarity-based misattributions, and this was aided by labels that supported recollection of headline veracity.

### Recollection benefitted more from fake news reminders than veracity labels

**Figure 4:**
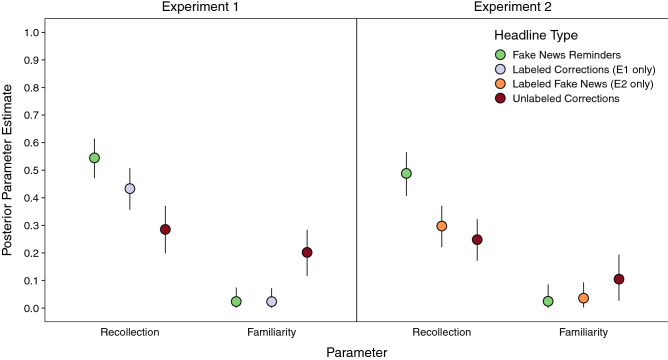
Latent parameter estimates for recollection and familiarity for each correction headline type condition. Points are posterior parameters estimated with MPT models, and error bars are 95% credibility intervals.

We formally examined the contributions of recollection- and familiarity-based retrieval to cued recall accuracy across correction methods (Fig. 4) using the MPT modeling approach explained previously (for a full description of this approach, see SI3). Recollection estimates when fake news reminders preceded corrections were credibly greater than for all other headline types in both experiments (smallest estimate = 0.11 [0.05, 0.17]). In addition, recollection estimates were credibly greater for labeled than unlabeled corrections (Experiment 1; estimate = 0.15 [0.08, 0.22]), but not credibly different for labeled fake news and unlabeled corrections (Experiment 2; estimate = 0.05 [− 0.02, 0.12]). As predicted, these differences paralleled the patterns for correct recall of real news. Familiarity estimates were generally low, but they were credibly greater for unlabeled corrections than all other corrections in Experiment 1 (estimate = 0.18 [0.09, 0.26]), but did not differ across conditions in Experiment 2 (i.e., CIs overlapped with 0). These results support the assertion that the memorial benefits conferred by fake news reminders and veracity-labeled corrections reflect larger contributions of recollection-based retrieval.

### Beliefs distinguished real from fake news more with reminders and labels

**Figure 5:**
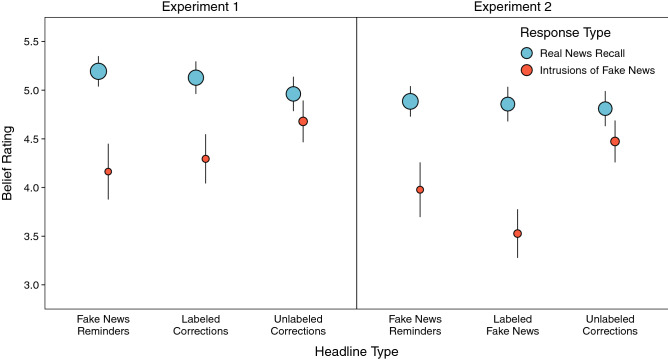
Beliefs in real news recall and intrusions of fake news for each correction headline type condition. Points are ratings estimated with mixed effects models, and error bars are 95% confidence intervals. Point sizes indicate for each cell the proportion of observations, which are also displayed in Supplementary Table S5.

We next examined differences in belief accuracy that were presumably based partly on memory differences across headline types (Fig. 5). Table 2 displays the complete model results for all belief rating analyses. We defined belief accuracy as the extent to which ratings were higher for real news recall and lower for intrusions of fake news. We deviated from our preregistered plan by including response type as a predictor instead of assessing each response type separately. Belief ratings were significantly higher for real news recall than intrusions of fake news in both experiments. Significant interactions qualified these differences. Experiment 1 showed significantly higher real news beliefs when fake news reminders had appeared than when corrections were unlabeled, *t*(774) = 3.12, *p* < 0.01, whereas beliefs in intrusions of fake news were significantly higher when corrections were unlabeled than for other corrections, smallest *t*(1322) = 2.81, *p* = 0.01. Experiment 2 showed no significant differences in real news beliefs, largest *t*(721) = 0.91, *p* = 0.64, and significantly lower beliefs in intrusions of fake news for labeled fake news than all other conditions, smallest *t*(1233) = 2.68, *p* = 0.02, and when fake news reminders had appeared than when corrections were unlabeled, *t*(1266) = 3.31, *p* < 0.01. These results show that, as for cued recall, fake news reminders and veracity labels improved belief accuracy. This conclusion is based on the consistent finding that the difference in belief ratings between real news recall and intrusions of fake news is substantially larger for fake news reminders and veracity-labeled headlines than unlabeled corrections, despite the inconsistency in the pairwise differences for real news recall between experiments. Together, these results suggest that belief accuracy depended partly on recollection of headlines and their veracity.

**Table 2:** Model results for beliefs in real news recall and intrusions of fake news in Phase 3.

Analysis	Effect	Experiment 1			Experiment 2		
		χ^2^	*df*	*p*	χ^2^	*df*	*p*
Overall	Response type	67.35	1	< 0.001	101.84	1	< 0.001
	Headline type	1.18	2	= 0.55	14.29	2	< 0.001
	Response type × headline type	21.75	2	< 0.001	32.83	2	< 0.001
Conditional real news recall	Headline type	4.49	2	= 0.11	0.25	2	= 0.88
	Classification	18.06	2	< 0.001	55.44	2	< 0.001
	Headline type × classification	14.33	4	< 0.01	4.56	4	= 0.34
Conditional intrusions of fake news	Headline type	2.61	2	= 0.27	12.82	2	< 0.01
	Classification	37.38	1	< 0.001	26.75	1	< 0.001
	Headline type × classification	3.15	2	= 0.21	4.43	2	= 0.11

**Figure 6:**
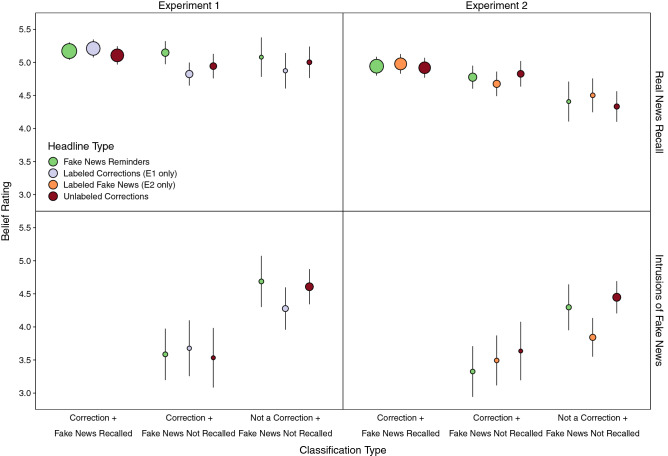
Belief ratings for real news recall and intrusions of fake news conditioned on correction classifications for each correction headline type condition. Points are ratings estimated with mixed effects models, and error bars are 95% confidence intervals. Point sizes indicate for each cell the proportion of observations, which are also displayed in Supplementary Table S5. Values are not displayed for intrusions for classified corrections when fake news was recalled due to sparse observations.

The results above correspond to the data visualized in Fig. 5 (for overall recall) and Fig. 6 (for conditional recall).

### Beliefs better distinguished real from fake news when corrections were remembered

We assessed the interplay of memory and beliefs further by conditioning beliefs on correction classifications (Fig. 6). Separate models were necessary for each response type because conditional analyses involving intrusions of fake news did not include accurately classified corrections for which fake news was recalled. Supplementary Table S5 shows the trial proportions. Experiment 1 revealed a significant interaction showing that belief ratings for real news details were consistently high across classifications, except that accurately classified corrections without fake news recall were associated with significantly higher beliefs when fake news reminders had appeared (middle green point) than when corrections were labeled (middle lavender point), *t*(2353) = 3.53, *p* < 0.01. Experiment 2 revealed a different pattern. Beliefs in recalled real news were significantly higher for accurately classified corrections with fake news recall than other classifications, smallest *z* ratio = 3.58, *p* < 0.001, and for accurately classified corrections without fake new recall than inaccurately classified corrections, *z* ratio = 4.09, *p* < 0.001. Moreover, both experiments showed that belief ratings for intrusions of fake news were significantly lower when corrections were accurately rather than inaccurately classified. These results show that remembering that headline details had been corrected was often associated with more accurate beliefs, especially for intrusions of fake news.

## General discussion

The present study examined the efficacy of reminder-based and veracity-labeling correction methods for improving memory and belief accuracy for news headlines. These comparisons were intended to identify roles for integration, conflict salience, and intentional forgetting during encoding as well as recollection and familiarity processes during retrieval. Presenting fake news reminders just before labeled corrections improved memory accuracy compared with only labeling corrections or fake news on its debut. Labeling corrections improved memory accuracy compared with presenting unlabeled corrections, whereas labeling fake news conferred no such benefit. Fake news reminders and veracity labels, especially when applied to fake news, both improved belief accuracy relative to unlabeled corrections. Retrieval process estimates and conditional analyses suggested that memory and belief accuracy were better when corrections were recollected. These results suggest that corrections promoting fake news remindings and memory for veracity labels differentially support recollection-based retrieval upon which beliefs about perceived headline accuracy are based.

The benefits conferred by fake news reminders and veracity-labeled corrections to memory and belief accuracy are compatible with the integration account of the continued influence effect^38^. This account proposes that retrieval of outdated information during new learning supports memory updating by promoting conflict saliency and the co-activation of the misinformation and its correction^3,15,16^. Support for this account comes from work showing that including misinformation reminders in narrative refutations improves event comprehension^16^ and inferential reasoning^15^. Additional support comes from work showing that fake news reminders^3^ and recall of fake news during corrections^34^ benefit memory and belief accuracy when corrections are recollected. The present findings add to this literature by suggesting that reminder-based and veracity-labeled corrections can promote integrative encoding to the extent that they trigger retrieval of fake news during real news corrections. The present results are also somewhat incompatible with the familiarity backfire prediction that repeating misinformation with corrections should lead misinformation to be more familiar and believable^36,39^. However, familiarity backfire was likely present in our results when corrections were not recollected. The present findings join the mounting evidence that familiarity backfire in aggregate results is elusive^12,13,40^ and provide more evidence for the nuanced interpretation that perceived accuracy is based more on familiarity when testing conditions undermine recollection of corrections^14^.

The present results are also relevant for disentangling the mechanisms of fake news reminder benefits. Prior work attributed such benefits to integrative encoding that supported recollection of misinformation, corrections, and their relationship^3^. However, fake news reminders always preceded veracity-labeled corrections, whereas the contrast condition included only unlabeled corrections. The confound between reminders and veracity labels created ambiguity for interpretation as reminder benefits could have reflected integrative encoding or conflict saliency^15^. We eliminated this confound by including veracity-labeled corrections without fake news reminders. Although veracity-labeled corrections improved memory and belief accuracy relative to unlabeled corrections, memory accuracy was greater when fake news reminders appeared. These findings suggest that previously observed reminder benefits reflected contributions of integrative encoding. However, the comparable benefits to belief accuracy of reminders and labels also suggests that recollection of veracity labels are salient cues upon which perceived accuracy is based.

Characterizing veracity label effects on belief accuracy is a focus of the nascent content labeling literature^4^. Prior work has shown that veracity labels are more effective at improving belief accuracy when they appear after rather than during or before fake news exposure^23^. Our study adds to this literature by showing the consequences for memory and belief accuracy of labeling fake news after exposure and labeling corrections during exposure. Both labels reduced intrusions of fake news and improved belief accuracy compared to when no labels appeared, but real news recall only benefitted when corrections were labeled. These asymmetrical effects suggest that labeling influences recollection of veracity that supports either selecting against false information or selecting for true information. This may explain why labeling fake news mitigated later intrusions but did not enhance recall of corresponding corrections. In this instance, instructions to disregard fake news made those headlines more distinctive, instead of less accessible, contrary to effects sometimes observed in intentional forgetting studies, in which people are instructed to remember some items and forget others^41^. To fully characterize veracity-labeling effects on various aspects of memory and beliefs, future studies should employ other arrangements of labeling, spacing, and repetitions. Studies should also include contextual information in labels, such as news sources and virality measures that provide social feedback (e.g., likes and shares).

Conditional analyses also suggested differences in the extent to which correction methods promoted integrative encoding that supported recollection. Differences in integrative encoding can be inferred from recall of outdated information and the extent to which it is positively associated with memory for updated details^21^. Here, positive associations between fake and real news recall provided evidence for integration. The memorial benefits associated with fake news recall were obtained more often when reminders and corrections were both labeled than when only real or fake news was labeled; these benefits were observed least for unlabeled corrections. This is compatible with the view that conditions that incite looking back to the past enable integrative encoding that supports recollection^42^. Here, reminders appeared to stimulate the most contact between phases, but veracity labels also served this function to a lesser extent. Converging evidence for recollection differences was shown in MPT model estimates as recollection paralleled assumed differences in integrative encoding across conditions.

The finding that recollection estimates were highest in the reminder conditions provides compelling evidence against the familiarity backfire prediction that reinstating fake news should increase the use of familiarity-based heuristics. In fact, familiarity estimates were highest for unlabeled corrections, which were least likely to reinstate fake news during corrections. The present findings align better with the possibility that during encoding, fake news reminders and veracity labels added cues to memory representations that supported recollection rejection^43–45^, which allowed participants to select real news and reject fake news when reporting. As mentioned previously, this may have also improved belief accuracy by allowing cues, such as veracity labels or memory for the relationship between real and fake news, to serve as a basis for judgments. This assertion is supported by the consistently lower beliefs in intrusions of fake news when participants also indicated remembering that fake news had been corrected.

### Limitations

As with all studies, the present one had limitations. One aim here was to remove the confounding effect of fake news reminders from the effects of labeling corrections to better account for the role of conflict saliency in correction effects on memory and belief accuracy. However, this does not fully isolate the fake news reminder effect because that would require a condition including fake news reminders alone (i.e., not followed by corrections). In addition, based on visual inspection of the data from both experiments, we decided to include in the analyses participants who failed our benchmark for attention-check performance. We mitigated any potential consequences of this by including in each model a by-participant random intercept effect of subjects to account for subsequent memory and belief effects of variability in attention during encoding. Finally, our participants were undergraduates from one university, thus precluding generalizability to the broader population. Future research in this area would benefit from replication attempts using nationally representative samples.

## Conclusion

The present study examined the effects of fake news reminders and veracity labels on subsequent memory for and beliefs in real and fake news headline details. Fake news reminders promoted high memory and belief accuracy, consistent with the integrative encoding view and contrary to the familiarity backfire view. Although veracity-labels also enhanced memory accuracy, such improvements were selective and never reached the level promoted by reminders. However, veracity labeling promoted high belief accuracy suggesting that memory for labels served as a cue for perceived accuracy. Memory and belief differences across corrections largely corresponded with differences in model-derived recollection estimates, which may have characterized the extent to which memory for corrections and associated details were used to select real news and reject fake news. A comprehensive and generalizable understanding of the effects of reminder-based and veracity-labeling correction methods will require examining the effects of moderating variables, such as source credibility, headline virality, and political concordance on memory, beliefs, and their relationship.

## Methods

All stimuli, data, and analysis scripts are available here: https://osf.io/zg8yx/. These experiments were approved by the Institutional Review Board at The University of North Carolina at Greensboro (UNCG) and were performed in accordance with relevant guidelines and regulations. Participants were recruited from UNCG, provided informed consent, and received course credit or a $15 gift card as compensation.

### Participants

The stopping rule for each experiment was to obtain usable data from at least 96 participants. These sample sizes match those from Wahlheim et al. (2020)^3^ and were sufficient to detect the smallest effects of interest according to power analyses based on that study for the sample in Experiment 1 (SI4) and on Experiment 1 for the sample in Experiment 2 (SI5). The final sample in each experiment included 96 participants (Experiment 1: 60 women, 34 men, 2 gender diverse ages 18–33 (*M* = 19.70, *SD* = 2.48); Experiment 2: 59 women, 34 men, 3 gender diverse ages 18–28 (*M* = 18.95, *SD* = 1.65)). In Experiment 1, data were excluded from 11 participants due to technical issues and one participant who was tested after reaching the target sample (108 participants were tested). In Experiment 2, data were excluded from 18 participants due to technical issues and one participant who was tested after reaching the target sample size (115 participants were tested). We deviated from our pre-registered plan to exclude participants based on failed attention checks and instead controlled for that variable in our analyses (for a detailed rationale, see SI6).

### Materials and design

Both experiments included 60 headline pairs from fact-checking websites (i.e., africacheck.org, bettergov.org, politifact.com, snopes.com, statesman.com) each comprising a real and fake news headline on the same unique topic. Fake news headlines included a false detail, and real news headlines included a true detail that corrected the false detail. All fake news headlines were originally portrayed by the media as being true. The headline format resembled breaking news updates on internet search engines. Real and fake news headlines about a topic appeared below an image related to the topic.

For counterbalancing, the 60 headline pairs were divided into four sets of 15 and rotated through the within-participant conditions; headline pairs appeared equally often in each condition across participants. Sets included comparable topic variety (i.e., politics, crime statistics, global warming, etc.) and distribution of qualitative and quantitative corrections. Qualitative corrections included changed sentence subjects. For example, the topic about the cause of Californian wildfires included the fake news detail that *bad forest management* was the cause, and the real news detail that *downed electric power lines* was the cause. In contrast, quantitative corrections included changed amounts. For example, the topic of the percentage of young women in Liberia who can read at 18 included the fake news detail that it was *less than 38 percent* and the real news detail that it was *around 60 percent*.

Experiment 1 used a within-participants design including a Headline Type variable (Repetition, Unlabeled Correction, Labeled Correction, Fake News Reminder + Labeled Correction). Experiment 2 used the same design, but the Labeled Fake News condition was substituted for the Labeled Correction condition. Each experiment included three phases. Phase 1 included 60 headlines (15 real news; 45 fake news). Phase 2 included 60 real news headlines. Phase 3 included a cued-recall test of the 60 headline topics. Each test item included the picture from the earlier-studied headline above an open-ended question about the detail that was corrected in Phase 2 when fake news had appeared in Phase 1.

### Procedure

The experimenter supervised data collection in groups of 1-4 participants using Zoom videoconferencing on a device other than the computer used for testing. Stimuli were presented electronically using E-Prime Go software^46^. In each phase, stimuli appeared in a fixed random order with the restriction that no more than three headlines from the same condition appeared consecutively. The average list position for each condition was equated to control for serial position effects.

Before Phase 1, participants were told that they would read real and fake news headlines and that they should study them for a later test. Each Phase 1 headline appeared twice each for 8000 ms followed by a 500 ms interstimulus interval (ISI). All 60 headlines appeared once in a first cycle before any headline repeated in a second cycle. On the first cycle, participants indicated their familiarity with each headline story from 1 (Definitely Unfamiliar) to 6 (Definitely Familiar). On the second cycle, they indicated their belief in each headline from 1 (Definitely False) to 6 (Definitely True). Each headline appeared 8000 ms followed by a rating prompt that appeared for 4000 ms. Headlines appeared without labels of their veracity for all items in Experiment 1. However, in the second cycle of Experiment 2, headlines in the Labeled Fake News condition appeared alone for the first 6000 ms and then with a message that the headline was false for the remaining 2000 ms. Participants were told to disregard or intentionally forget these items.

Before Phase 2, participants were told that they would read real news headlines. They were also told that some would repeat real news from Phase 1 and others would correct fake news from Phase 1. They were also told about the experimental conditions and to note when headlines were corrections. Each Phase 2 headline appeared once for 8000 ms (+ 500 ms ISI), including fake news reminders that preceded real news corrections. Headlines in the Repetition, Unlabeled Correction, and Labeled Fake News (Experiment 2 only) conditions appeared without labels of their relationship to headlines in Phase 1. In contrast, headlines in the Labeled Correction (Experiment 1 only) and Fake News Reminder + Labeled Correction conditions appeared with labels indicating whether they corrected or repeated fake news.

Before Phase 3, participants were told that they would recall real news details from Phase 2, indicate if the headlines had corrected fake news, and if so, recall the corrected fake news details from Phase 1 (in that order). They were told that they would also rate their beliefs in the real news details that they recalled from Phase 2. Test cues appeared above a text box until participants typed their recall responses. After attempting to recall the real news detail from Phase 2, participants rated their belief that what they recalled was true in reality from 1 (Definitely False) to 6 (Definitely True) in Phase 3. They then indicated whether the real news in Phase 2 had corrected fake news in Phase 1 by pressing 1 (Yes) or 0 (No). After responding “yes,” they were prompted to recall the Phase 1 fake news headline. Note that, unlike the previous phases, the cued recall test was self-paced to avoid placing time pressure on the three possible responses given during each trial.

After Phase 3, participants completed a seven-item cognitive reflection test that consisted of a reworded version of the original three-item task from^47^ and a four-item non-numeric task from^48^. Test scores were the number of questions answered correctly. We report the rationale for including this measure and the results of these exploratory analyses including responses from this measure in SI7.3.

### Ethics approval and consent to participate

This experiment was approved by the Institutional Review Board of the University of North Carolina at Greensboro. All participants gave informed consent.

## Supplementary Information

Supplementary Information.

## Data Availability

The stimuli and de-identified data can be downloaded from the Open Science Framework: https://osf.io/zg8yx/.

## References

[1] Pennycook G, Rand DG (2021). The psychology of fake news. Trends Cogn. Sci..

[2] Roozenbeek J (2020). Susceptibility to misinformation about COVID-19 around the world. R. Soc. Open Sci..

[3] Wahlheim CN, Alexander TR, Peske CD (2020). Reminders of everyday misinformation statements can enhance memory for and beliefs in corrections of those statements in the short term. Psychol. Sci..

[4] Morrow G, Swire-Thompson B, Polny JM, Kopec M, Wihbey JP (2022). The emerging science of content labeling: Contextualizing social media content moderation. J. Assoc. Inf. Sci. Technol..

[5] Johnson HM, Seifert CM (1994). Sources of the continued influence effect: When misinformation in memory affects later inferences. J. Exp. Psychol. Learn. Mem. Cogn..

[6] Wilkes AL, Leatherbarrow M (1988). Editing episodic memory following the identification of error. Q. J. Exp. Psychol. Sect. A.

[7] Ecker UKH, Lewandowsky S, Tang DTW (2010). Explicit warnings reduce but do not eliminate the continued influence of misinformation. Mem. Cognit..

[8] Lewandowsky S, Ecker UKH, Seifert CM, Schwarz N, Cook J (2012). Misinformation and its correction: continued influence and successful debiasing. Psychol. Sci. Public Interest.

[9] Schwarz, N., Sanna, L. J., Skurnik, I. & Yoon, C. Metacognitive Experiences and the intricacies of setting people straight: Implications for debiasing and public information campaigns. In *Advances in Experimental Social Psychology* vol. 39 127–161 (Elsevier, 2007).

[10] Cook J, Bedford D, Mandia S (2014). Raising climate literacy through addressing misinformation: Case studies in agnotology-based learning. J. Geosci. Educ..

[11] Pluviano S, Watt C, Ragazzini G, Della Sala S (2019). Parents’ beliefs in misinformation about vaccines are strengthened by pro-vaccine campaigns. Cogn. Process..

[12] Swire-Thompson B, DeGutis J, Lazer D (2020). Searching for the backfire effect: Measurement and design considerations. J. Appl. Res. Mem. Cogn..

[13] Swire-Thompson B, Miklaucic N, Wihbey JP, Lazer D, DeGutis J (2022). The backfire effect after correcting misinformation is strongly associated with reliability. J. Exp. Psychol. Gen..

[14] Swire B, Ecker UKH, Lewandowsky S (2017). The role of familiarity in correcting inaccurate information. J. Exp. Psychol. Learn. Mem. Cogn..

[15] Ecker UKH, Hogan JL, Lewandowsky S (2017). Reminders and repetition of misinformation: Helping or hindering its retraction?. J. Appl. Res. Mem. Cogn..

[16] Kendeou P, Walsh EK, Smith ER, O’Brien EJ (2014). Knowledge revision processes in refutation texts. Discourse Process..

[17] Kendeou P, Butterfuss R, Kim J, Van Boekel M (2019). Knowledge revision through the lenses of the three-pronged approach. Mem. Cognit..

[18] Putnam AL, Wahlheim CN, Jacoby LL (2014). Memory for flip-flopping: Detection and recollection of political contradictions. Mem. Cognit..

[19] Stadtler M, Scharrer L, Brummernhenrich B, Bromme R (2013). Dealing with uncertainty: Readers’ memory for and use of conflicting information from science texts as function of presentation format and source expertise. Cogn. Instr..

[20] Wahlheim CN, Jacoby LL (2013). Remembering change: The critical role of recursive remindings in proactive effects of memory. Mem. Cognit..

[21] Wahlheim, C. N., Garlitch, S. M. & Kemp, P. L. Context differentiation and remindings in episodic memory updating. In *Psychology of Learning and Motivation* vol. 75 245–277 (Elsevier, 2021).

[22] Swire-Thompson B (2021). Correction format has a limited role when debunking misinformation. Cogn. Res. Princ. Implic..

[23] Brashier NM, Pennycook G, Berinsky AJ, Rand DG (2021). Timing matters when correcting fake news. Proc. Natl. Acad. Sci..

[24] Bäuml K-H, Pastötter B, Hanslmayr S (2010). Binding and inhibition in episodic memory—Cognitive, emotional, and neural processes. Neurosci. Biobehav. Rev..

[25] Sahakyan, L., Delaney, P. F., Foster, N. L. & Abushanab, B. List-method directed forgetting in cognitive and clinical research. In *Psychology of Learning and Motivation* vol. 59 131–189 (Elsevier, 2013).

[26] Smith SM, Vela E (2001). Environmental context-dependent memory: A review and meta-analysis. Psychon. Bull. Rev..

[27] Jacoby LL (1998). Invariance in automatic influences of memory: Toward a user’s guide for the process-dissociation procedure. J. Exp. Psychol. Learn. Mem. Cogn..

[28] Jacoby LL (1991). A process dissociation framework: Separating automatic from intentional uses of memory. J. Mem. Lang..

[29] Jacoby LL (1999). Ironic effects of repetition: Measuring age-related differences in memory. J. Exp. Psychol. Learn. Mem. Cogn..

[30] Evans J, Stanovich K (2013). Dual-process theories of higher cognition: Advancing the debate. Perspect. Psychol. Sci..

[31] Kahneman, D. *Thinking Fast and Slow*. (Macmillan, 2011).

[32] Pennycook G, Fugelsang JA, Koehler DJ (2015). What makes us think? A three-stage dual-process model of analytic engagement. Cognit. Psychol..

[33] Ecker UKH, O’Reilly Z, Reid JS, Chang EP (2020). The effectiveness of short-format refutational fact-checks. Br. J. Psychol..

[34] Kemp PL, Alexander TR, Wahlheim CN (2022). Recalling fake news during real news corrections can impair or enhance memory updating: The role of recollection-based retrieval. Cogn. Res. Princ. Implic..

[35] Sanderson, J. A. & Ecker, U. K. H. The challenge of misinformation and ways to reduce its impact. In *Handbook of Learning from Multiple Representations and Perspectives* (eds. Van Meter, P., List, A., Lombardi, D. & Kendeou, P.) 461–476 (Routledge, 2020). 10.4324/9780429443961-30.

[36] Skurnik I, Yoon C, Schwarz N (2007). Education About Flu Can Reduce Intentions to Get a Vaccination.

[37] Wahlheim CN, Smith WG, Delaney PF (2019). Reminders can enhance or impair episodic memory updating: A memory-for-change perspective. Memory.

[38] Ecker UKH (2022). The psychological drivers of misinformation belief and its resistance to correction. Nat. Rev. Psychol..

[39] Schwarz N, Newman E, Leach W (2016). Making the truth stick & the myths fade: Lessons from cognitive psychology. Behav. Sci. Policy Wash..

[40] Lewandowsky, S., Cook, J. & Lombardi, D. *Debunking handbook 2020*. (2020) 10.17910/B7.1182.

[41] Sahakyan L, Foster NL (2009). Intentional forgetting of actions: Comparison of list-method and item-method directed forgetting. J. Mem. Lang..

[42] Jacoby LL, Wahlheim CN, Kelley CM (2015). Memory consequences of looking back to notice change: Retroactive and proactive facilitation. J. Exp. Psychol. Learn. Mem. Cogn..

[43] Brainerd CJ, Reyna VF, Wright R, Mojardin AH (2003). Recollection rejection: False-memory editing in children and adults. Psychol. Rev..

[44] Gallo DA (2004). Using recall to reduce false recognition: Diagnostic and disqualifying monitoring. J. Exp. Psychol. Learn. Mem. Cogn..

[45] Moore KN, Lampinen JM (2016). The use of recollection rejection in the misinformation paradigm: Recollection rejection of misinformation. Appl. Cogn. Psychol..

[46] Psychology Software Tools. [E-Prime Go]. (2020).

[47] Frederick S (2005). Cognitive reflection and decision making. J. Econ. Perspect..

[48] Thomson KS, Oppenheimer DM (2016). Investigating an alternate form of the cognitive reflection test. Judgm. Decis. Mak..

