# Supplementary material for: Fake news reminders and veracity labels differentially benefit memory and belief accuracy for news headlines
Source: Sci Rep. 2022 Dec 17;12:21829. doi: 10.1038/s41598-022-25649-6 (PMC9758464; doi:10.1038/s41598-022-25649-6)
Supplement: Supplementary file 1 — Supplementary Information. [file 41598_2022_25649_MOESM1_ESM.docx]

**Supplementary Information**

**1. Statistical Methods**

All analyses were conducted using R software^1^. We examined the effects of interest using logistic and linear mixed-effects models from *lme4*^2^. The models included fixed effects of Headline Type and Correction Classifications, where applicable. The models also included by-participant and by-item random intercepts. We performed Wald’s χ^2^ hypothesis tests using the *Anova* function of the *car* package^3^ and post-hoc comparisons controlling for multiple comparisons using the Tukey method in the *emmeans* package^4^. The complete model specifications are available in the analysis scripts on the OSF: (<https://osf.io/zg8yx/>). The significance level was α = .05.

**2. Phase 1 Ratings**

***2.1 Familiarity Ratings in Phase 1***

Table S1 (top row) displays the baseline familiarity ratings in Phase 1. In both experiments, participants perceived real news headlines as more familiar than fake news headlines in the reminder and unlabeled correction headlines, smallest *z* ratio = 2.82, *p* = .02. Familiarity ratings did not differ between real news headlines and fake news headlines associated with veracity labels in each experiment, largest *z* ratio = 1.38, *p* = .51. In both experiments, there were no significant differences in familiarity ratings across fake news headlines in the three correction conditions, largest *z* ratio = 1.86, *p* = .25.

***2.2 Baseline Belief Ratings in Phase 1***

Table S1 (bottom row) displays the baseline belief ratings in Phase 1. In both experiments, participants believed real news headlines more than fake news headlines in all three correction conditions, smallest *z* ratio = 5.25, *p* < .001. In Experiment 1, beliefs in fake news did not differ across the correction conditions, largest *z* ratio = 0.49, *p* = .96. In Experiment 2, including veracity-labels with fake news led to lower belief ratings than for all other conditions, smallest *z* ratio = 63.92, *p* < .001, showing that participants attended to the labels when making their ratings. Belief in fake news did not differ between the other two correction conditions, *z* ratio = 0.58, *p* = .94, replicating Experiment 1.

**3. Multinomial Process Tree Analyses of Cued Recall**

We fitted hierarchical Bayesian multinomial processing tree (MPT) models to the cued recall data for each experiment using the TreeBUGS package^5^. MPT models are a class of measurement models that are used to estimate the probability of latent cognitive parameters from the frequency of observed categorical data. Here, the categorical data refer to the three possible cued recall response types: correct recall of real news from Phase 2, intrusions of fake news from Phase 1, and other types of intrusion errors. All ambiguous cued recall responses and responses to repeated real news headlines were not considered.

MPT models assume discrete cognitive states that lead participants to make one of these types of responses. Here, we used the independence model used extensively in similar research^e.g., 6–8^. Figure S1 shows the model. This model assumes that recollection (*Pr*) can lead to correct recall of the real news headline. In the absence of recollection (1 – *Pr*), the fake news and real news headlines may both be familiar (*Pf*), in which case participants may guess randomly between headlines with equal probability, leading to either correct real news recall (*Gc* = 0.5) or intrusions of fake news (1 – *Gc*). Finally, in the absence of familiarity (1 – *Pf*), responses reflect random guessing with an equal probability between the correct response and incorrect fake news possibilities (*Gr* = 0.5) or an unrelated response (1 – *Gr*).

The hierarchical Bayesian MPT approach is advantageous because it accounts for heterogeneity between participants in the parameters while also affording a clear interpretation of null differences among conditions. The TreeBUGS package was used to fit the model with four Markov-Chain Monte-Carlo samples each comprising 100,000 iterations with 2,000 used for warm-up, 20,000 used for adaptation, and with a thinning factor of five. We confirmed good convergence between the chains both visually and by checking that the *Ȓ* statistic (i.e., the ratio of between-chain variance to within-chain variance) was close to one for all the parameters of the fitted models. We also ensured appropriate model fit by verifying that the *ppp* values were greater than .05.

To draw inferences about the effect of headline type on recollection and familiarity, we made within-subjects comparisons that computed the difference between the mean group posterior of one condition and that of another. Table S2 shows the posterior differences between the conditions. When CIs do not overlap with 0, we consider the difference credible. The analysis script to run or reproduce the model, as well as related information such as the model priors and its results, can be found on the OSF.

**4. Power Analysis for Experiment 1**

When planning Experiment 1, we used a standardized effect size estimate from the smallest effect of interest in Wahlheim et al. (2020)^9^ as a basis for a power analysis. That small-medium effect (*dz* = .44) corresponded to the finding that belief accuracy for misinformation intrusions was significantly greater when corrections had appeared with reminders rather than alone. At the time, our tool of choice for conducting power analyses was G*Power 3.1.9.2^10^. According to G*Power a sample size of 43 participants was sufficient to detect that effect size with 80% power (alpha = .05). However, our primary hypothesis about the effects of labeled corrections on overall memory and belief accuracy in the present study was that such corrections should lead to intermediate values relative to labeled corrections with fake news reminders and unlabeled corrections that appeared alone. We reasoned that those effect sizes may be smaller than the smallest effect size of interest from Wahlheim et al.^9^ We therefore chose to match the larger sample size from Wahlheim et al. (*N* = 96), which, according to G*Power, would allow us to detect a smaller effect size (*dz* = .29) with 80% power (alpha = .05). In the next section, a simulation analysis of Experiment 1 in the present study verifies that this sample size was more than sufficient to detect the smallest observed effect size of interest.

**5. Sensitivity Analyses**

***5.1. Experiment 1 Sensitivity Analysis / Power Analysis for Experiment 2***

We conducted a simulation-based sensitivity analysis using R software^1^ based on the results from Experiment 1, which also served as a power analysis for planning the sample size in Experiment 2. The smallest effect size of interest in Experiment 1 corresponded to the difference in correct real news recall in Phase 3 between veracity-labeled corrections alone and those following fake news reminders. To examine the sensitivity to detect this effect, we first calculated the odds ratio of the pairwise difference between these conditions. To do this, we modeled the effects of experimental manipulations in Experiment 1 on real news recall in Phase 3 using a logistic mixed effects model, fitted with the *glmer* function from *lme4*^2^. We included the Headline Type predictor as a fixed effect and included by-participant and by-item random intercepts. We then conducted a significance test (z test) to derive the log odds ratio effect size for the difference between the conditions of interest using the *dotest* function from *simr*^11^. We converted the log odds ratio to an odds ratio using the *exp* function from *base R* and interpreted the effect size using the *interpret_oddsratio* function from the *effectsize* package^12^. The odds ratio for effect size of interest was small (*OR* = 1.68^13^).

We conducted a simulation-based sensitivity analysis using *simr*^11^ to examine the power to detect the odds ratio from the comparison described above. A sensitivity analysis based on 1,000 simulations with alpha set at .05 revealed that with 97 participants, Experiment 1 had 99.90% [*95% CI* = 99.44, 100.00] power to detect a small effect (*OR* = 1.68). To further determine the sample size necessary to detect this effect we generated power curve showing power levels across varying sample sizes. The power curve below (see Figure S2) shows that based on 1,000 simulations with 80% power (alpha = .05), a sample size of approximately 25 participants was sufficient required to detect an *OR* = 1.68. Experiment 1, which included 96 participants, was thus well powered for the smallest effect of interest.

***5.2. Experiment 2 Sensitivity Analyses***

Similar to Experiment 1, to examine the sensitivity to detect the smallest effect size of interest, we conducted a simulation-based sensitivity analysis in the same manner as for Experiment 1. In Experiment 2, the smallest effect of interest was the difference in fake news recall in Phase 3 between for the conditions with veracity-labeled fake news in Phase 1 and fake news reminders before veracity-labeled headlines in Phase 2. This effect was of interest because it assessed differences in correction types on real news recall. A sensitivity analysis based on 1,000 simulations with alpha set at .05 revealed that with a sample size of 96 participants, Experiment 2 had 100% [*95% CI* = 99.63, 100.00] power to detect a small effect (*OR* = 2.12). A power curve based on 1,000 simulations with 80% power and alpha set at .05 (Figure S3) indicated that a sample size of 16 participants would have been sufficient to detect an effect of this size. Experiment 2 was therefore well-powered to detect the smallest effect of interest.

**6. Attention Checks**

We pre-registered a plan to exclude participants who failed more than two attention checks. However, the data collection rate was far slower than we anticipated given the virtual collection method. To meet our pre-registered sample size of at least 96 participants, we decided to include all participants in the analyses, regardless of their performance on the attention checks. For failing two attention checks, we would have excluded 11 participants in Experiment 1 and 10 participants in Experiment 2. Visual inspection of the data showed that although participants who we would have excluded sometimes performed poorly, many of them performed comparably to the other participants and vice versa. We therefore concluded that participants who failed more than two attention checks were unlikely to be categorically different from the others. To the extent that differences in task engagement was associated with memory and beliefs, we accounted for that by including the frequency of passed attention checks as a fixed effect (regressor of non-interest) in the mixed effects models. Moreover, reliable participant-level differences were captured in the by-participant random intercepts of the models.

**7. Exploratory Analyses**

In our pre-registrations, we outlined several exploratory questions motivated by previous findings^14,15^. However, upon further reflection, we decided to constrain our report to focus only on the exploratory questions that were most relevant to our overarching goals. Additional exploratory analyses that were not included in the manuscript or the present document can be found here: <https://osf.io/zg8yx/>. Below, we discuss findings from exploratory analyses concerning how 1) belief accuracy changes for fake news headlines in Phase 1 that eventually produced intrusions in Phase 3 (SI 7.1) and 2) whether patterns of cued recall performance remain constant when only considering responses that people believed were true (SI 7.2). We also tested whether analytic thinking that serves to correct faulty intuitions^16,17^ co-varies with memory and belief accuracy when recalling corrections of fake news using a variant of the cognitive reflection test^18,19^ as an individual differences measure (SI 7.3).

***7.1 Comparing Belief Ratings in Phases 1 and 3***

Based on previous work showing that prior exposure to fake news increases perceived accuracy^15^, it is possible that presenting fake news in Phase 1 and again in Phase 2 (along with the correction headline that repeats features of the fake news headline) results in greater overall belief in fake news at test relative to baseline beliefs in the current study. To assess this, we compared belief ratings during Phases 1 and 3 across all the correction conditions for instances when fake news intruded on the cued recall test (see Table S3). Experiment 1 showed that fake news was perceived as more believable in Phase 3 than in Phase 1, smallest *t* ratio = 3.97, *p* < .001. In Experiment 2, there was a significant interaction, χ^2^ (2) = 159.58, *p* < .001, showing that fake news was perceived as more believable in Phase 3 than Phase 1 when such headlines were labeled as false in Phase 1, *t* ratio = 15.03, *p* < .001. There were no differences in belief ratings for fake news when corrections were unlabeled or fake news reminders preceded labeled corrections in Phase 2, largest *t* ratio = 1.34, *p* = .18.

***7.2. Memory Accuracy for Responses Perceived as True***

***Correct Real News Recall***

We examined whether the cued recall pattern changed when only considering responses perceived as true following across experiment comparisons manipulating report criterion based on believability^9^. A model was fitted to correct real news recall with Headline Type as a factor for corrected headlines that were perceived as true at test (see Figure S4). Parallel to the findings in the main text, Experiment 1 showed significantly higher real news recall for fake news reminder headlines than when corrections appeared with or without veracity-labels, smallest *z* ratio = 5.01, *p* < .001. Additionally, labeling corrections led to significantly higher real news recall than unlabeled corrections, *z* ratio = 3.55, p = .001. Experiment 2 showed significantly higher real news recall when fake news reminders immediately preceded corrections (*M* = .68 [*95% CI* = .57, .77]) regardless of whether fake news headlines appeared with veracity labels in Phase 1 before being corrected by unlabeled real news in Phase 2, smallest *z* ratio = 7.09, *p* < .001. In contrast to the main text, real news recall was higher when fake news appeared with a veracity label in Phase 1 (*M* = .50 [*95% CI* = .38, .61]) than unlabeled corrections headlines in Phase 2 (*M* = .43 [*95% CI* = .32, .55]), *z* ratio = 2.63, *p* = .02. This discrepancy indicated that guessing biases differed based on whether participants perceived their responses as true or false. More work is needed to understand this difference.

***Intrusions of Fake News***

A model was fitted to fake news intrusion errors with Headline Type as a factor for corrected headlines that were perceived as true at test (see Figure S4). Consistent with the main text, both experiments showed that corrections appearing with veracity labels, regardless of whether fake news reminders appeared in Phase 2 led to lower intrusion rates than presenting corrections without veracity labels (Experiment 1: *M* = .10 [*95% CI* = .07, .14]; Experiment 2: *M* = .12 [*95% CI* = .09, .16]), smallest *z* ratio = 4.61, *p* < .001. There were no significant differences in intrusions depending on whether fake news reminders were provided (Experiment 1: *M* = .04 [*95% CI* = .03, .06]; Experiment 2: *M* = .05 [*95% CI* = .04, .07]), corrections featured veracity-labels (*M* = .05 [*95% CI* = .04, .08]), or fake news appeared with veracity-labels in Phase 1 (*M* = .07 [*95% CI* = .05, .10]), smallest *z* ratio = 2.07, *p* = .10.

***7.3. Cognitive Reflection and its Association with Memory and Belief Accuracy***

Analytic thinking may be associated with memory and belief accuracy based on work reporting positive associations between the number of correct responses on cognitive reflection tests and discernment between real and fake news headlines^20^. One possibility is that individual differences in recollection corresponds with the use of analytic thinking to overcome the pull of fake news familiarity during memory and belief judgments. This is consistent with the view from a dual-process theory that analytic thinking serves to correct faulty intuitions^16,17^. Using a seven-item cognitive reflection test, we examined how strongly cognitive reflection was associated with memory and belief accuracy. We correlated cognitive reflection, measured as the number of correct responses on the cognitive reflection test, with correct recall of real news, intrusions of fake news, and truth discernment for these responses computed as belief ratings for real news recall minus intrusions of fake news for each participant. Note that there were fewer observations for truth discernment because not all participants produced both responses at least once.

Figure S5 shows that cognitive reflection was positively associated with real news recall, *r*(192) = .42, *p* < .001, negatively associated with intrusions of fake news, *r*(192) = -.26, *p* < .001, and was not associated with truth discernment, *r*(172) = -.01, *p* = .86. These results are partially consistent with the view that analytic thinking serves to correct faulty information, but the absence of its association with truth discernment implies a more complex relationship among analytic thinking, memory, and beliefs. Differences in perceived accuracy that co-vary with analytic thinking may reflect memory differences upon which beliefs are sometimes based. However, more work is needed to understand the cognitive differences underlying truth discernment given concerns about whether the cognitive reflection test measures analytic thinking ability per se^21,22^.

**References**

1. R Core Team. R: A language and environment for statistical ## computing. R Foundation for statistical computing. (2021).

2. Bates, D., Mächler, M., Bolker, B. & Walker, S. Fitting linear mixed-effects models using lme4. *J. Stat. Softw.* **67**, (2015).

3. Fox, J. & Weisberg, S. *An R companion to applied regression*. (Sage, 2019).

4. Lenth, R. emmeans: Estimated marginal means, aka least- squares means. (2021).

5. Heck, D. W., Arnold, N. R. & Arnold, D. TreeBUGS: An R package for hierarchical multinomial-processing-tree modeling. *Behav. Res. Methods* **50**, 264–284 (2018).

6. Bartsch, L. M., Singmann, H. & Oberauer, K. The effects of refreshing and elaboration on working memory performance, and their contributions to long-term memory formation. *Mem. Cognit.* **46**, 796–808 (2018).

7. Jacoby, L. L. Ironic effects of repetition: Measuring age-related differences in memory. *J. Exp. Psychol. Learn. Mem. Cogn.* **25**, 3–22 (1999).

8. Loaiza, V. M. & Srokova, S. Semantic relatedness corrects the age-related binding deficit in working memory and episodic memory. *J. Gerontol. Ser. B* **75**, 1841–1849 (2020).

9. Wahlheim, C. N., Alexander, T. R. & Peske, C. D. Reminders of everyday misinformation statements can enhance memory for and beliefs in corrections of those statements in the short term. *Psychol. Sci.* **31**, 1325–1339 (2020).

10. Faul, F., Erdfelder, E., Buchner, A. & Lang, A.-G. Statistical power analyses using G*Power 3.1: Tests for correlation and regression analyses. *Behav. Res. Methods* **41**, 1149–1160 (2009).

11. Green, P. & MacLeod, C. J. SIMR : an R package for power analysis of generalized linear mixed models by simulation. *Methods Ecol. Evol.* **7**, 493–498 (2016).

12. Ben-Shachar, M., Lüdecke, D. & Makowski, D. Effectsize: Estimation of effect size indices and standardized parameters. *J. Open Source Softw.* **5**, 2815 (2020).

13. Chen, H., Cohen, P. & Chen, S. How big is a big odds ratio? Interpreting the magnitudes of odds ratios in epidemiological studies. *Commun. Stat. - Simul. Comput.* **39**, 860–864 (2010).

14. Wahlheim, C. N., Garlitch, S. M. & Kemp, P. L. Context differentiation and remindings in episodic memory updating. in *Psychology of Learning and Motivation* vol. 75 245–277 (Elsevier, 2021).

15. Pennycook, G., Cannon, T. D. & Rand, D. G. Prior exposure increases perceived accuracy of fake news. *J. Exp. Psychol. Gen.* **147**, 1865–1880 (2018).

16. Evans, J. & Frankish, K. *In two minds: Dual processes and beyond*. (Oxford University PressOxford, 2009). doi:10.1093/acprof:oso/9780199230167.001.0001.

17. Stanovich, K. E. *The robot’s rebellion: finding meaning in the age of Darwin*. (University of Chicago Press, 2004).

18. Frederick, S. Cognitive reflection and decision making. *J. Econ. Perspect.* **19**, 25–42 (2005).

19. Thomson, K. S. & Oppenheimer, D. M. Investigating an alternate form of the cognitive reﬂection test. *Judgm. Decis. Mak.* **11**, 15 (2016).

20. Pennycook, G. & Rand, D. G. The psychology of fake news. *Trends Cogn. Sci.* **25**, 388–402 (2021).

21. Pennycook, G. & Rand, D. G. Lazy, not biased: Susceptibility to partisan fake news is better explained by lack of reasoning than by motivated reasoning. *Cognition* **188**, 39–50 (2019).

22. Sinayev, A. & Peters, E. Cognitive reflection vs. calculation in decision making. *Front. Psychol.* **6**, (2015).

**Table S1**

*Familiarity and Belief Ratings*

|  | Experiment 1 | | | | | Experiment 2 | | | |
| --- | --- | --- | --- | --- | --- | --- | --- | --- | --- |
|  |  |  |  |  |  | |  |  |  |
| Measure | Real News Repetition | Reminder +  Correction | Labeled  Correction | Unlabeled  Correction | Real News Repetition | | Reminder +  Correction | Labeled  Fake News | Unlabeled  Correction |
|  |  |  |  |  |  | |  |  |  |
|  |  |  |  |  |  | |  |  |  |
| Familiarity | 3.02 [2.77, 3.27] | 2.85 [2.60, 3.10] | 2.95 [2.70, 3.20] | 2.87 [2.62, 3.12] | 2.93 [2.70, 3.16] | | 2.76 [2.53, 2.99] | 2.82 [2.59, 3.05] | 2.74 [2.51, 2.97] |
| Baseline Beliefs | 3.88 [3.69, 4.08] | 3.53 [3.34, 3.73] | 3.53 [3.33, 3.72] | 3.51 [3.32, 3.70] | 4.21 [4.06, 4.36] | | 4.00 [3.85, 4.15] | 1.42 [1.28, 1.57] | 3.98 [3.83, 4.12] |

*Note*: Bootstrap 95% confidence intervals are displayed in brackets.

**Table S2**

*Parameter Estimates of Recollection and Familiarity*

|  | Experiment 1 | | | Experiment 2 | | |
| --- | --- | --- | --- | --- | --- | --- |
|  |  |  |  |  |  |  |
|  | Mean [95% CIs] Posterior Differences | | | Mean [95% CIs] Posterior Differences | | |
|  |  |  |  |  |  |  |
|  |  |  |  |  |  |  |
| Parameter | Reminder + Corrections vs.  Labeled Corrections | Reminder + Corrections vs.  Unlabeled Corrections | Labeled Corrections  vs.  Unlabeled Corrections | Reminder + Corrections vs.  Labeled Fake News | Reminder + Corrections vs.  Unlabeled Corrections | Labeled Fake News  vs.  Unlabeled Corrections |
|  |  |  |  |  |  |  |
|  |  |  |  |  |  |  |
| Recollection | **.11 [.05, .17]** | **0.26 [.19, .40]** | **.15 [.08, .22]** | **.20 [.12, .25]** | **.24 [.17, .31]** | .05 [-.02, .12] |
| Familiarity | .00 [-.05, .06] | **-.18 [-.27, -.09]** | **-.18 [-.27, -.09]** | -.01 [-.08, .06] | -.08 [-.18, .01] | -.07 [-.17, .02] |

*Note.* CI = credibility interval. Bold values indicate credible differences in the posterior differences.

**Table S3**

*Belief Ratings for Headlines that Produced Intrusions of Fake News in Phases 1 and 3*

|  | Experiment 1 | | | Experiment 2 | | |
| --- | --- | --- | --- | --- | --- | --- |
|  |  |  |  |  |  |  |
| Phase | Reminder +  Corrections | Labeled  Corrections | Unlabeled  Corrections | Reminder +  Corrections | Labeled  Fake News | Unlabeled  Corrections |
|  |  |  |  |  |  |  |
|  |  |  |  |  |  |  |
| Phase 1 | 3.90 [3.58, 4.22] | 3.79 [3.49, 4.09] | 3.86 [3.59, 4.13] | 4.03 [3.77, 4.29] | 1.66 [1.42, 1.90] | 4.17 [3.96, 4.39] |
| Phase 3 | 4.17 [3.85, 4.49] | 4.02 [3.72, 4.32] | 4.38 [4.11, 4.65] | 3.83 [3.57, 4.09] | 3.62 [3.39, 3.86] | 4.25 [4.04, 4.47] |

*Note*: Bootstrap 95% confidence intervals are displayed in brackets.

**Table S4**

*Proportions of Correction Classifications on the Cued Recall Test in Phase 3*

|  | Experiment 1 | | | Experiment 2 | | |
| --- | --- | --- | --- | --- | --- | --- |
|  |  |  |  |  |  |  |
| Classification Type | Reminder +  Corrections | Labeled  Corrections | Unlabeled  Corrections | Reminder +  Corrections | Labeled  Fake News | Unlabeled  Corrections |
|  |  |  |  |  |  |  |
|  |  |  |  |  |  |  |
| Correction +  Fake News Recalled | .58 | .49 | .44 | .50 | .40 | .36 |
| Correction +  Fake News Not Recalled | .29 | .29 | .26 | .30 | .30 | .26 |
| Not a Correction +  Fake News Not Recalled | .14 | .22 | .30 | .20 | .30 | .39 |

*Note.* The values above correspond to the cell sizes of conditional real news recall and intrusions of fake news in Figure 3.

**Table S5**

*Proportions of Real News Recall and Intrusions of Fake News on the Cued Recall Test in Phase 3*

|  | Experiment 1 | | | | Experiment 2 | | |
| --- | --- | --- | --- | --- | --- | --- | --- |
|  |  |  |  |  | |  |  |
| Response by Classification Type | Reminder +  Corrections | Labeled  Corrections | Unlabeled  Corrections | Reminder +  Corrections | | Labeled  Fake News | Unlabeled  Corrections |
|  |  |  |  |  | |  |  |
|  |  |  |  |  | |  |  |
| Real News Recall (Overall) | (.64) | (.56) | (.50) | (.60) | | (.46) | (.44) |
| Correction + Fake News Recalled | .50 | .42 | .37 | .42 | | .31 | .30 |
| Correction + Fake News Not Recalled | .11 | .11 | .09 | .15 | | .11 | .09 |
| Not a Correction + Fake News Not Recalled | .03 | .03 | .04 | .03 | | .04 | .05 |
|  |  |  |  |  | |  |  |
| Intrusions of Fake News (Overall) | (.08) | (.10) | (.16) | (.10) | | (.13) | (.16) |
| Correction + Fake News Recalled | .00 | .00 | .00 | .01 | | .01 | .00 |
| Correction + Fake News Not Recalled | .04 | .03 | .03 | .04 | | .04 | .03 |
| Not a Correction + Fake News Not Recalled | .04 | .07 | .13 | .05 | | .08 | .13 |
|  |  |  |  |  | |  |  |

*Note.* The values above correspond to the cell sizes of beliefs ratings in Figure 5 (overall) and Figure 6 (conditioned on classification type). Intrusions of fake news < .01 are displayed as .00 for continuity with the rounding scheme in the other cells.

**Figure S1**

*Independence Model*

*Note.* Independence multinomial model that estimates familiarity and recollection for responses in the cued recall task. R represents recollection of real news headlines and F represents familiarity of real news headlines. *Correct recall real news* are responses that included correct details from Phase 2 headlines. *Intrusions fake news* are responses that included details from false details from Phase 1 headlines. *Other* are responses that included details that were inconsistent with either correct or fake news headlines as well as omissions.

**Figure S2**

*Experiment 1 Power Curve*

*Note.* Power curve to detect a very small effect (*OR* = 1.68) as a function of sample size (number of levels in Subject) using data from Experiment 1.

**Figure S3**

*Experiment 2 Power Curve*

**

*Note.* Power curve to detect a small effect (*OR* = 2.12) as a function of sample size (number of levels in Subject) using data from Experiment 2.

**Figure S4**

*Cued Recall of Real and Fake News Details in Phase 3 as a Function of Perceived Truth*

**

*Note.* Probabilities of real news recall and intrusions of fake news conditioned on perceived truth at test for each correction headline type condition. Points are probabilities estimated from mixed effects models; errors bars are 95% confidence intervals.

**Figure S5**

*Cognitive Reflection and its Associations with Real News Recall, Intrusions of Fake News, and Truth Discernment in Beliefs in Cued Recall Responses*

*
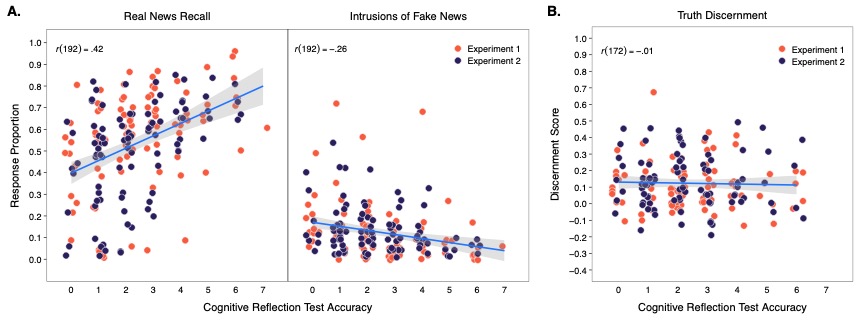
*

*Note.* The scatterplots display the association between cognitive reflection test accuracy and real news recall, intrusions of fake news, and truth discernment for each individual subject. The colored points represent individual participants from each experiment. Shaded regions are 95% confidence intervals around regression lines fitted to the data from both experiments.
